# Abnormal intra-thoracic fat distribution in patients with metabolic syndrome with and without myocardial infarction

**DOI:** 10.1186/1532-429X-13-S1-P166

**Published:** 2011-02-02

**Authors:** Umjeet S Jolly, Matthew Brymer, Nowell Fine, Charles McKenzie, Tisha Joy, Maria Drangova, James A White

**Affiliations:** 1 University of Western Ontario, London, ON, Canada; 2 Biomedical Imaging Research Centre (BIRC), London, ON, Canada; 3 London Health Sciences Centre, London, ON, Canada

## Introduction

Adipose distribution is a useful tool for cardiovascular risk stratification when compared to body mass index (BMI) alone. We sought to compare intra-thoracic fat distribution in patients with or without metabolic syndrome (MetS), and to identify differences in those with and without evidence of prior myocardial infarction (MI), as determined by delayed enhancement (DE) MRI.

## Methods

A total of 110 consecutive patients referred for DE MRI suspected of having either MetS or prior MI were enrolled and received a thoracic fat survey using HASTE imaging. This was performed throughout the full thorax in the sagittal plane at 10mm intervals (10mm slice thickness, zero gap) during shallow breathing. Off-line signal threshold analysis (CMR42, Circle Cardiovascular Inc. Calgary) was used to determine intra-thoracic fat volume using a >10 SD signal threshold above the mean of 4 skeletal muscle sample volumes. Total intra-thoracic fat volume was indexed to body surface area to provide a measure of relative fat distribution. Patients were stratified into 3 disease groups according to stringent criteria; A) MetS+/MI-, B) MetS-/MI+, and C) MetS+/MI+. MetS was defined as any 3 of the following 5 criteria: BMI > 30 kg/m^2^, serum triglycerides > 1.7 mmol/L, HDL-C <1.0 mmol/L for men or HDL-C <1.3 mmol/L for women, HgbA1c >6.5%, SBP >130 mmHg or DBP >85 mmHg. MI was defined as the presence of subendocardial-based DE in a coronary artery distribution. 16 volunteers were also imaged to provide a normal reference cohort. Data was analyzed using ANOVA statistical analysis.

## Results

**Figure 1:**
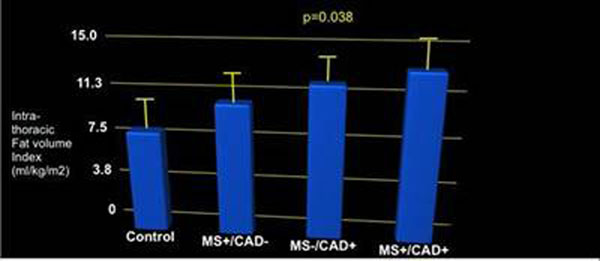
Indexed intra-thoracic fat volume including BMI. There is an incremental increase of indexed intra-thoracic fat volume with MetS and prior MI.

In total 80 of the 110-screened patients met disease criteria. All 16 volunteers completed the imaging protocol (total N=96). Mean age was 59.8±12.5 years (78% male, 71% Caucasian). 26 patients (32%) were identified as MetS+/MI-, 33 (41%) as MetS-/MI+, and 21 (26%) as MetS+/MI+. All 16 volunteers were MetS-/MI-. The intra-thoracic fat distribution was significantly different among the groups, as follows; MetS+/MI+ was 13.3 ± 2.0 ml/kg/m^2^, MetS-/MI+ was 12.15 ± 2.06 ml/kg/m^2^, MetS+/MI- was 10.57 + 2.37 ml/kg/m^2^ and controls were 8.16 ± 2.29 ml/kg/m^2^ (p value = 0.038) (See Figure 1).

## Conclusion

Indexed intra-thoracic fat volume is elevated relative to healthy controls in patients with either MetS or prior MI, and more significantly elevated in patients with both MetS and MI. This may be an important variable for the prediction of ischemic cardiovascular events, especially in patients with MetS. Prospective outcomes-based cohort studies are warranted to explore this potential.

